# How Is the World Responding to the Novel Coronavirus Disease (COVID-19) Compared with the 2014 West African Ebola Epidemic? The Importance of China as a Player in the Global Economy

**DOI:** 10.4269/ajtmh.20-0135

**Published:** 2020-03-11

**Authors:** Elisa M. Maffioli

**Affiliations:** Department of Health Management and Policy, School of Public Health, University of Michigan, Ann Arbor, Michigan

## Abstract

This article describes similarities and differences in the response of governments and the international community to the current 2019 coronavirus disease (COVID-19) and the 2014 West African Ebola epidemic. It expresses the opinion that the speed and scale of the response to COVID-19 are affected by the important role that China plays in the global economy. By contrast, insufficient and less timely action was initially undertaken in West African countries during the 2014 Ebola epidemic. It concludes by stating why preparedness for and response to all disease outbreaks, also in countries of lower economic importance, should become a priority in the global health agenda.

Respiratory illness caused by a new coronavirus (the 2019 novel coronavirus disease [COVID-19]) was first identified in Wuhan city, Hubei Province, China, on December 8, 2019 and then reported to the public on December 31, 2019.^1^ As of March 9, 2020, the virus continues to cause outbreaks at alarming rates. Spreading to 25 countries in less than 2 months, the virus has now affected more than 109,000 people and caused more than 3,800 deaths in 105 countries.^2^ The current situation resembles what the world encountered in the last decade with other unknown viruses—the 2009–2010 swine flu (H1N1), the 2014 West African Ebola virus disease (EVD), the 2018–2020 Democratic Republic of Congo (DRC) EVD, and the 2015–2016 Zika virus outbreak in Latin America. How does the governments’ and the international community’s response to the COVID-19 contrast that to the 2014 West African Ebola virus epidemic?

The COVID-19 shares similarities with the 2014 EVD. Preliminary investigation suggests a zoonotic origin^3^; there is a lack of knowledge about the epidemiology of the virus^4^; and there is widespread misinformation, generating panic and mistrust among the public. Estimates put the fatality rate of COVID-19 at less than 3% and its basic reproduction number (R0) between 2 and 3,^5^ compared with the high fatality rate of the 2014 EVD, typically estimated to be around 50–70%, and an R0 of a similar range.^6^ Whereas economic impacts of the 2014 EVD were estimated at $25.2 billion,^7^ a recent assessment on COVID-19 suggests that the disease could cost the global economy $1.1 trillion in lost income.^8^ Because of the forced quarantine of Hubei Province, there is a common understanding that the economic costs will be considerable, as businesses, schools, and factories have been closed for weeks. As China is the second largest global economy, with 19% of share (GDP: $13.4 trillion),^9^ the costs will be likely larger than that of the last health epidemic in the country (the 2002–2003 severe acute respiratory syndrome [SARS]), which costed the global economy $40 billion.^10^ Because of the integrated international supply chain, several countries are facing a slowdown, and prices for metals, oil, or other materials have also fallen on expectations of lower demand. Even the technology sector and the global fashion industry are feeling the impacts.

However, the Chinese government has taken prompt actions, such as shutting down Wuhan’s Huanan Seafood Market on January 1, 2020; building a 1,000-bed hospital in 10 days; and putting cities on lockdown. Data on the genome were deposited in a public repository, providing researchers with the opportunity to develop diagnostic tests, treatments, or vaccines. The international community also reacted quickly. The WHO declared COVID-19 a public health emergency of international concern (PHEIC) on January 30, 2020. The Trump administration unprecedentedly barred entry to Chinese nationals who had been in China in the previous 14 days. The United States, along with several other countries, imposed entry screenings at airports, and British Airways even suspended flights. More than 20 countries organized prompt evacuation of their nationals. International agencies are shipping supplies. Although media coverage has been massive since the first month,^11^ both the Chinese government and the international community are taking further steps to curb misinformation. The WHO very recently announced that a vaccine could be available in 18 months.

By contrast, the potentially catastrophic consequences of the 2014 EVD were initially downplayed by the African countries and the international community. West African governments were unprepared, the initial response was slow and insufficient, and it was concentrated when the peak of the epidemic had already passed. International health workers were evacuated and hospitals temporarily shut down. Only when the WHO declared the PHEIC in August 2014—5 months after it received information about the virus—did resources pour in. The WHO was criticized for its failure to warn the world in time and for making decisions about global health security only as a result of mounting political pressures.^12^

The Chinese government has been criticized for the early missteps in the response.^13^ It took about 3 weeks to inform the public and bring awareness of the potential outbreak of a highly contagious disease. There was an attempt to control the flow of information and censor doctors’ announcements of the true number of cases. It took nearly 2 weeks of requests from the WHO to have a team of medical experts travel to China to assist in investigating the virus. Despite this initial public health cover-up, its response represents a remarkable progress compared with how it handled the 2002–2003 SARS pandemic.^14^ If anything, the actions taken, together with the openness in reporting about COVID-19, are buying the rest of the world some precious time. On the one hand, better preparedness through strengthening of the public health system—for example, the China CDC after the 2002–2003 SARS epidemic—is helping the Chinese government respond to this novel virus in a timely way. On the other hand, however, global economic and geopolitical interests and the role of the Chinese economy worldwide might contribute to why the international community’s attention and response stand out when compared with the 2014 EVD, or even with the 2018–2020 DRC EVD.

As the world continues to change with increasing population and people living in proximity to animals, there will be new and re-emerging pathogens that will appear and spread. There is urgency for governments in low-income countries, such as those in sub-Saharan Africa, to invest in improving their fragile health systems and for donors to support their preparedness. It is essential that the international community be more alert and engaged in a timelier way—as it is currently for COVID-19—to defeat future health threats affecting countries of lower economic status. As Dr. Thedros Adhanom Ghebreyesus, the director-general of WHO, said in a statement on February 11, 2020, “This outbreak is a test of solidarity—political, financial and scientific.” The speed and large scale of response to COVID-19 should be set as examples for future disease outbreaks. The WHO and international donors are now helping African countries to prepare for this novel virus^15^ as the first case was registered in Egypt on February 14, 2020 and new cases were recorded more recently in Algeria, Senegal, South Africa, Cameroon, Nigeria and Togo. Therefore, preparedness for and response to all disease outbreaks should become a priority for the global health agenda.
